# Antibiotic Use in China’s Public Healthcare Institutions During the COVID-19 Pandemic: An Analysis of Nationwide Procurement Data, 2018–2020

**DOI:** 10.3389/fphar.2022.813213

**Published:** 2022-02-14

**Authors:** Ying Yang, Xin Geng, Xiaojun Liu, Xiaotong Wen, Ruonan Wu, Dan Cui, Zongfu Mao

**Affiliations:** ^1^ School of Public Health, Wuhan University, Wuhan, China; ^2^ Global Health Institute, Wuhan University, Wuhan, China; ^3^ Department of Health Management, School of Public Health, Fujian Medical University, Fuzhou, China; ^4^ Dong Fureng Economic and Social Development School, Wuhan University, Wuhan, China

**Keywords:** antibiotic consumption, antimicrobial resistance, COVID-19, rational drug use, China

## Abstract

**Background:** The overuse of antibiotics is a serious public health problem and a major challenge in China, and China lacks up-to-date evidence on the nationwide antibiotic use in different healthcare settings. The changes of China’s antibiotic use under the COVID-19 pandemic are still unknown.

**Objective:** This study aimed to investigate the use of antibiotics in China’s public medical institutions based on a three-year nationwide surveillance and to examine the impact of the COVID-19 pandemic on China’s antibiotic consumption.

**Methods:** This study used nationwide drug procurement data from the China Drug Supply Information Platform (CDSIP). We retrospectively analyzed antibiotic procurement data of 9,176 hospitals and 39,029 primary healthcare centers (PHCs) from 31 provinces in mainland China from January 2018 to December 2020. Antibiotic utilization was measured by defined daily doses (DDDs) and DDD per 1,000 inhabitants per day (DID). Generalized linear regression models were established to quantify the impact of the COVID-19 pandemic on antibiotic use.

**Results:** The total antibiotic consumption among all healthcare settings increased from 12.94 DID in 2018 to 14.45 DID in 2019, and then dropped to 10.51 DID in 2020. More than half of antibiotics were consumed in PHCs, especially in central regions (59%–68%). The use of penicillins (J01C) and cephalosporins (J01D) accounted for 32.02% and 28.86% of total antibiotic consumption in 2020. During 2018–2020, parenteral antibiotics accounted for 31%–36% of total antibiotic consumption; the proportion is more prominent in central and western regions and the setting of hospitals. Access category antibiotics comprised 40%–42% of the total utilization. Affected by COVID-19, the antibiotic consumption was significantly dropped both in hospitals (*β* = −.11, *p* < .001) and PHCs (*β* = −.17, *p* < .001), as well as in total (*β* = −.14, *p* < .001). Significant increments were observed in the proportion of total antibiotics (*β* = .02, *p* = .024) consumed in hospitals (against the consumption in all healthcare settings), as well as parenteral antibiotics (*β* = 1.73, *p* = .001).

**Conclusion:** The consistent preferred use of penicillin and cephalosporin, as well as injections, among China’s public healthcare institutions should draw concern. China’s antibiotic consumption significantly declined during the COVID-19 pandemic, which brings opportunities for antibiotic use management in China.

## 1 Introduction

Antimicrobial resistance (AMR) has been increasingly concerned as a major public health problem worldwide, which affects all areas of health and incurs high economic costs to society (WHO, 2016). It was estimated that AMR could cause 10 million deaths per year by 2050 if no action is taken to stop its spread (O'Neill, 2016), and up to 3.5 billion US dollars will be spent annually on average due to AMR in Europe, North America, and Australia (OECD, 2018). The G20 Hangzhou Summit in 2016, as well as the 71st United Nations (UN) General Assembly, recognized AMR as a global priority health issue, as it cannot be single-handedly managed or mitigated by any organization or nation (WHO, 2019).

China is one of the countries that consume the most antibiotics and have one of the highest prevalence of AMR in the world, and the average antibiotic consumption of China in 2013 was six times higher than that of the United States and Europe (Zhang et al., 2015). To address this issue, the Chinese government had taken many measures to strengthen antimicrobial management, for example, the introduction of guidance for clinical use of antibiotics, the establishment of national surveillance networks for both antibiotic use and resistance, and the implementation of “Special Rectification Activities of Clinical Use of Antibacterial” (Xiao et al., 2013; Liu et al., 2019; Yin et al., 2019). However, these policies and strategies did not fully achieve the expected results. In China, inappropriate antibiotic prescribing was highly prevalent nationwide (51.4%), and over half of the antibiotic prescriptions were inappropriate in secondary-level and tertiary-level hospitals (Zhao et al., 2021).

In recent years, a large number of studies explored the patterns and trends of antibiotic consumption in China, both nation-level studies (Wushouer et al., 2017; Wushouer et al., 2020; Liu et al., 2021; Qu et al., 2018; Chen et al., 2018) and province-level studies, for example, in Shanghai (Lin et al., 2016), Hubei (Zhang et al., 2019), Shaanxi (Xu et al., 2020), and Shandong (Yin et al., 2018; Song et al., 2020; Yin et al., 2021). The abovementioned studies found that the overall antibiotic consumption demonstrated an upward trend, and the use proportion of broad-spectrum and parenteral antibiotics was high. In China, the increased use of last-resort antibiotics has attracted public health concerns, and China’s antibiotic use is far from the global target set up by the WHO. With the continuous advancement and deepening of China’s medical reform, we still need updated nationwide evidence to present the current situation of China’s antibiotic consumption.

The aggressive COVID-19 pandemic has stressed health systems around the world. The European Centre for Disease Prevention and Control (ECDC) reported that most likely as a result of the COVID-19 pandemic, the total antibiotic consumption decreased by more than 15% between 2019 and 2020 in most EU/EEA (European Union and European Economic Area) countries, mostly in primary care (ECDC, 2021). The unprecedented decrease in community antibiotic consumption noted for all groups of antibiotics in nearly all EU/EEA countries is the largest in ESAC-Net (European Surveillance of Antimicrobial Consumption Network)’s two-decade long antimicrobial consumption surveillance history, and one example of the far-reaching consequences of the COVID-19 pandemic (Hogberg et al., 2021). Evidence from Singapore revealed that the antimicrobial utilization in hospitals significantly reduced by 361.46 DDD/1000 patient days/month before and after the peak of the COVID-19 pandemic (Ng et al., 2021). In the United States, nationwide studies reported the substantial decline of antibiotic prescribing of outpatients during the COVID-19 pandemic (Buehrle et al., 2021; King et al., 2021). These findings from EU/EEA countries, Singapore, and the United States consistently mentioned that the significant reduction in the use of systemic antibiotics during the COVID-19 pandemic might be attributed to infection control measures and reduced contact with the health service (Swedres–Svarm, 2020; Blix and Høye, 2021; Buehrle et al., 2021; ECDC, 2021; Gagliotti et al., 2021; Hogberg et al., 2021; King et al., 2021; Ng et al., 2021; Penalva et al., 2021).

In China, the substantial decline in medical services was still observed due to strict COVID-19 prevention and control measures. According to the government statistics, affected by the COVID-19 pandemic, the total medical services in China’s medical institutions declined dramatically in 2020, with the decrement of 11.2% in clinical visits and 13.5% in hospitalizations compared with 2019 (National Health Commission, 2021). In 2020, drugs used in China’s hospitals (expressed in defined daily doses) decreased by 18.2% in the first quarter and 11.5% in the second quarter as compared with the corresponding periods in 2019; and for antibiotics, the use proportion of anti-infective drugs dropped by 1.3 percentage points (from 6.5% to 5.2%) in the first half of 2020 compared with the corresponding period in 2019 (Chinese Pharmaceutical Association, 2020). The previous study also mentioned that the decrease in the number of patients seen and treated under the COVID-19 pandemic has led to a significant reduction in prescriptions for antibiotics in China’s primary healthcare centers (PHCs) (Zhang et al., 2021). Under the context of the COVID-19 pandemic, the antibiotic consumption in Chinese medical institutions might have changed; however, no one to our knowledge has described the changes in antibiotic use during the COVID-19 pandemic in China.

Therefore, this study aimed to provide the latest evidence on the use of antibiotics in China’s public medical institutions based on a three-year nationwide surveillance. In addition, we exploratorily estimated the change of antibiotic consumption in China’s public medical institutions under the shock of the COVID-19 pandemic at the population level. The findings of this study may be useful in monitoring the increase in drug resistance and developing appropriate use interventions, as well as providing baseline information for future assessment and regional comparison.

## 2 Materials and Methods

### 2.1 Data Sources

Data used in the present study were obtained from the China Drug Supply Information Platform (CDSIP), which is a comprehensive information platform for national drug procurement data. The CDSIP was constructed and operated by the Statistical Information Center of the National Health Commission of the People’s Republic of China, and was officially launched on October 22, 2015. Since then, health facilities upload information daily on ordering, storing, delivering, and medicine settlement to the CDSIP; the government can monitor prices, quantities, distribution, and warehousing, and can organize medications purchased by medical institutions and strengthen management based on relevant information. Thus, the CDSIP database covered drug procurement order data of all provincial drug centralized procurement platforms from 31 provinces (autonomous regions and municipalities) in mainland China.

In 2015, the Chinese government required that all public medical institutions should purchase all drugs to be used through the provincial-level drug centralized procurement platform (General Office of the State Council, 2015), which to a great extent ensured the integrality and accuracy of the CDSIP procurement data. It was estimated that the CDSIP covered more than 80% of drug purchasing data from national health facilities in mainland China (Liu et al., 2021).

### 2.2 Data Collection and Management

In this study, we collected procurement data of 48,205 public medical institutions of 31 provinces included in the CDSIP, in which 9176 public hospitals and 39,029 PHCs were involved (Table 1). According to the number of public hospitals in each province pronounced in the China Health Statistics Yearbook (National Bureau of Statistics, 2020), public hospitals covered by the CDSIP accounts for 76.92% of the total number of public hospitals in mainland China. Regional variation in antibiotic use was calculated by dividing the country into three regions (eastern China, central China, and western China) based on the economic zone division criteria of the China Statistical Yearbook (National Bureau of Statistics, 2020). Medical institutions were divided into tertiary hospitals, secondary hospitals, and PHCs. The procurement records covered a 3-year study period from January 1, 2018 to December 31, 2020. However, for 7 provinces (Beijing, Guangdong, Hunan, Yunnan, Tibet, Chongqing, and Sichuan), their procurement data in 2020 were incomplete in the CDSIP database. Thus, only 24 provinces were involved when it comes to 3-year annual trend analysis.

**TABLE 1:** Distribution of sample medical institutions.

Region^a^	Provinces	Sample medical institutions			%^b^
		Hospitals	PHCs	Total	
Eastern China	Tianjin	93	225	318	65.96
	Hebei	527	1999	2,526	75.39
	Liaoning	371	1220	1,591	83.75
	Shanghai	153	403	556	91.07
	Jiangsu	322	1609	1,931	70.31
	Zhejiang	348	1231	1,579	77.85
	Fujian	221	1141	1,362	80.66
	Shandong	518	1908	2,426	64.59
	Hainan	91	366	457	66.91
	Beijing^c^	191	1856	2047	88.02
	Guangdong^c^	595	1441	2036	80.95
Central China	Shanxi	439	95	534	93.40
	Jilin	237	846	1,083	88.43
	Heilongjiang	484	1165	1,649	82.88
	Anhui	258	1484	1742	71.87
	Jiangxi	287	1574	1861	84.91
	Henan	585	2125	2,710	84.66
	Hubei	269	1295	1,564	68.45
	Hunan^c^	378	2081	2,459	77.94
Western China	Guangxi	287	1368	1,655	85.16
	Guizhou	208	1028	1,236	73.50
	Shaanxi	372	1636	2008	82.67
	Gansu	249	1522	1771	84.41
	Qinghai	101	337	438	90.99
	Ningxia	64	338	402	96.97
	Xinjiang	230	943	1,173	48.42
	Inner Mongolia	248	1157	1,405	73.16
	Yunnan^c^	287	1440	1727	68.17
	Tibet^c^	37	—	37	31.62
	Chongqing^c^	157	897	1,054	68.56
	Sichuan^c^	569	4,298	4,867	81.40
Total		9,176	39029	48205	76.92

a Classification of the regions was obtained from the China Health Statistics Yearbook.

b Percentage was calculated by dividing the number of sampled public hospitals by the total number of public hospitals in the region.

c For the 7 provinces, their procurement data in 2020 were incomplete in the CDSIP database. Thus, data of the above 7 provinces were only involved in part of the analysis content.

PHCs: primary healthcare centers.

The procurement data extracted from the CDSIP database include the name of the medical institution, procurement date, drug YPID (Yao Pin Identifier) code, drug generic name, dosage form, specification, package, manufacturer, price per unit, purchasing unit (by box, bottle, or branch), purchase volume, and purchase expenditures. We collected procurement data of antibiotics according to the Anatomical Therapeutic and Chemical (ATC) classification J01 (i.e., antibacterial for systemic use). A total of 178 unique chemical substance names were identified in single or combination antibiotics. These antibiotics were aggregated into 10 ATC-3 groups. Included antibiotic drugs were dichotomized into two categories (oral and parenteral) according to the route of administration and were divided into four groups (access, watch, reserve, and other) based on WHO’s “Access, Watch, Reserve” (AWaRe) category (WHO, 2017). In addition, we considered the use of antibiotics in several major classes: J01C penicillins, J01D cephalosporins, J01F macrolides/lincosamides, and J01M quinolones (Zhang et al., 2019).

### 2.3 Outcome Measures

The procurement volume of antibiotics was measured based on its defined daily dose (DDD), which is developed by the WHO to compare drug consumptions and refers to the average maintenance dose per day for a drug used for its main indication in adults (WHO Collaborating Centre for Drug Statistics Methodology, 2020). In this study, the DDD of the drugs which could not be coded in WHO’s ATC/DDD Index 2021 system was determined based on the dosage regimen recommended in the manufacturers’ instructions, as approved by the China Food and Drug Administration.

The procurement data were then converted into the DDD per 1000 inhabitants per day (DID) at the level of the active substance. As there was no direct access to the exact number of population covered by the sample facilities, we calculated the weighted population as a proxy based on Eq. 1. This calculation process was under two assumptions: (a) no significant difference existed in the distribution of the sample facilities, and (b) there was no significant difference in the distribution of the population covered by the sample hospitals across the provinces (Wushouer et al., 2020).

$$Y_{i}=\sum_{i=1}^{31}P_{i}\times \frac{n_{i}}{N_{i}}\times \frac{m_{i}}{M_{i}},$$ (1)

where Y_*i*_ refers to the coverage inhabitants in a given year in province *i*, P_*i*_ refers to the total population in a given year in province *i*, n_*i*_ refers to the number of sample facilities in province *i*, N_*i*_ refers to the number of total public health facilities in province *i*, m_*i*_ refers to the number of inpatients and outpatients in sample facilities in province *i*, and M_*i*_ refers to the number of inpatients and outpatients in all public health facilities in province *i*. All the relevant census data for calculating inhabitants were collected from the China Health Statistics Yearbook (National Health Commission, 2020) and the China Statistics Year Book (National Bureau of Statistics, 2020).

### 2.4 Statistical Analysis

We first applied the descriptive statistical method to quantify the patterns and trends of antibiotic consumption, which were expressed in the epidemiological distribution characteristics of antibiotic consumption in different years, geographic regions, healthcare settings, and drug categories. Descriptive statistics such as mean, percentage, and growth rate were applied.

The generalized linear model (GLM) was used to examine the change of health facilities’ antibiotic consumption under the impact of the COVID-19 pandemic. According to previous studies, the impact of the COVID-19 pandemic on the use of antibiotics in medical institutions might be related to the drug treatment of COVID-19 cases (Li et al., 2020; Langford et al., 2021) and the decline of medical services due to COVID-19 control measures (Buehrle et al., 2021; ECDC, 2021; Hogberg et al., 2021; King et al., 2021; Ng et al., 2021). Thus, two variables (medical service decline and number of COVID-19 cases under treatment) were selected as the indirect measures of the COVID-19 pandemic to estimate the change of antibiotic use at the population level.

In the first model, we first divided the twenty-five sample provinces into three groups according to their decline range of medical services (1, <15%; 2, 15%–25%; 3, ≥25%) in the first half of 2020 as compared with the corresponding periods in 2019 (Supplementary Table S1). The change of each outcome variable was compared among three province groups between the pre- and post-COVID-19 pandemic periods, and the regression model was constructed as follows:

$$Y_{ijt}=\alpha_{0}+\beta \times {\text{Time}}_{ijt}\times {\text{Group}}_{ijt}+\gamma \times {\text{X}}_{ijt}+\varepsilon_{ijt}.$$ (2)

In the second model, the monthly number of COVID-19 cases under treatment was estimated, and three intervals were divided (0, no cases under treatment; 1, 1–49 cases; 2, 50–199 cases; and 3, ≥200 cases) (Supplementary Table S2). The regression model was constructed as follows:

$$Y_{ijt}=\alpha_{0}+\beta \times {\text{Time}}_{ijt}\times Case_{ijt}+\gamma \times {\text{X}}_{ijt}+\varepsilon_{ijt}.$$ (3)

In Eqs 2, 3 above, Y indicates the dependent variables, that is, the volume (expressed in the DDD) of antibiotics and the use proportion; *i* indicates a specific antibiotic; *j* represents the province; and *t* indicates the month (36-month periods). Time_ijt_ is a time dummy variable, where pre-COVID-19 (January 2018 to December 2019) is 0 and post-COVID-19 (January to December 2020) is 1. Group_ijt_ and Case_ijt_ are treatment dummy variables for indirect impact degree of COVID-19 in each province. Time_ijt_×Group_ijt_ and Time_ijt_×Case_ijt_ are interaction terms between the time dummy variable and treatment dummy variable, and the regression coefficient *β* of Time_ijt_×Group_ijt_ or Time_ijt_×Case_ijt_ reflects the impact of the COVID-19 pandemic on antibiotic consumption. X_ijt_ is a series of covariates potentially affecting antibiotic use, including coverage inhabitants of each province, per capita gross domestic product (GDP) of each province (Zhen et al., 2021), and potential seasonality effect (Chandy et al., 2014). ɛ_ijt_ refers to the random error term.

Modified Park tests and Box-Cox tests were used to estimate family distribution and link function of each outcome indicator (Zhang et al., 2017; Li et al., 2021). Based on the results (Supplementary Table S3), we used gamma distribution and log link for most of the outcome variables; in addition, gamma distribution and identity link, Gaussian distribution and identity link, and Gaussian distribution and log link were also applied for other dependent variables. Data were managed and analyzed in Microsoft Excel 2019 and Stata 15.0 (Stata Corp LP, College Station, TX, USA). A difference with *p* < .05 was considered to indicate statistical significance.

## 3 Results

### 3.1 Overall Antibiotic Use

**FIGURE 1:**
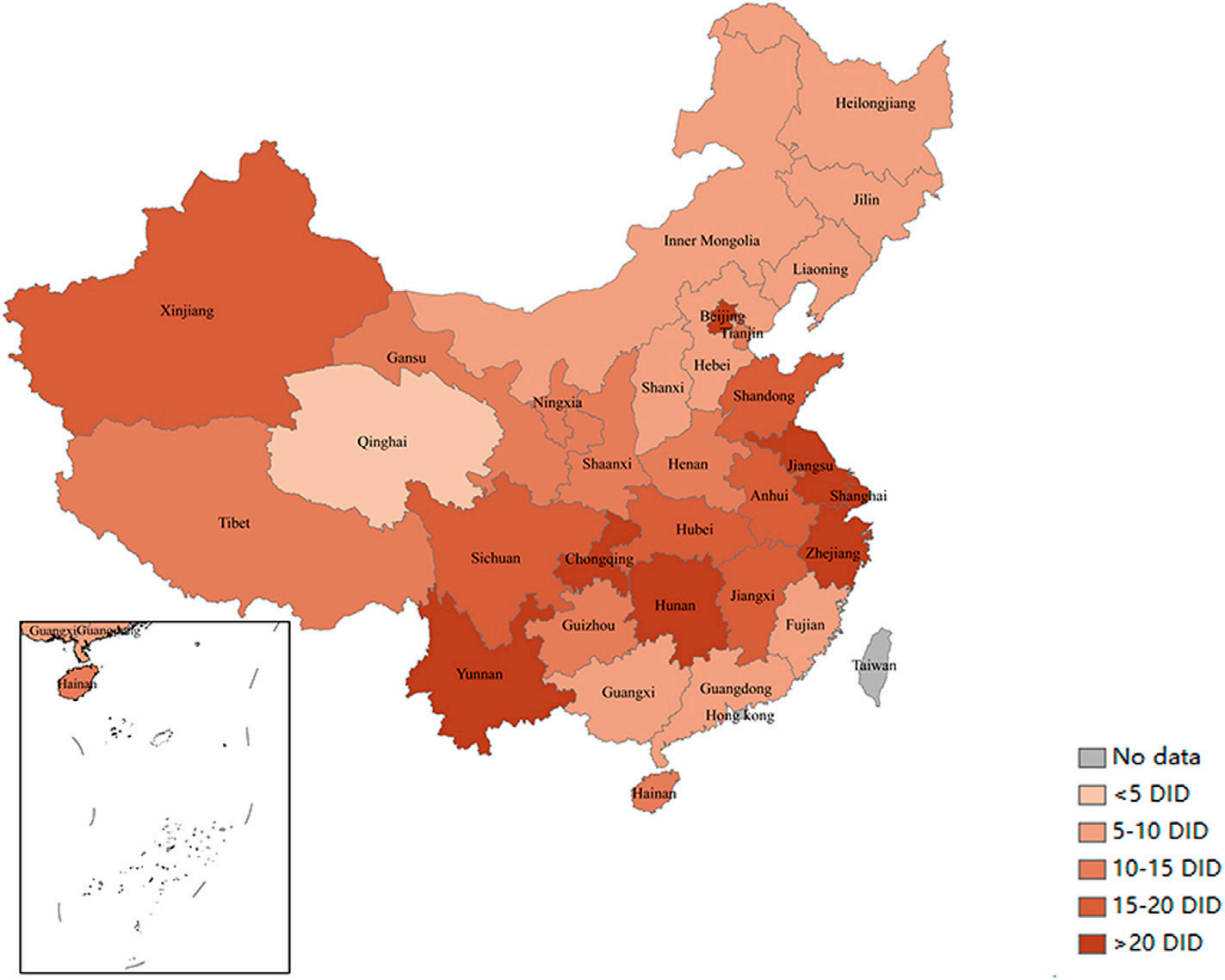
Antibiotic consumption (expressed in DID) in China’s public medical institutions in 2019. Note: Data of 31 provinces were involved. DID, defined daily doses per 1,000 inhabitants per day.

Figure 1 presents the antibiotic consumption (expressed in DID) of public medical institutions of 31 provinces in mainland China. In 2019, Beijing, Shanghai, Jiangsu, Zhejiang, Yunnan, Hunan, and Chongqing ranked top in antibiotic consumption, while Qinghai, Heilongjiang, and Jilin ranked the lowest.

As shown in Table 2, the total antibiotic consumption increased from 12.94 DID in 2018 to 14.45 DID in 2019 with a 11.65% increment, and antibiotics consumed in eastern, central, and western regions increased by 11.18%, 16.25%, and 5.05%, respectively. In 2020, the total antibiotic consumption (10.51 DID) dropped 27.25% as compared with 2019; the decrement of 27.38%, 28.54%, and 23.86% was observed in eastern, central, and western regions. During 2018–2019, the consumption of oral antibiotics increased from 8.92 DID to 9.30 DID (4.25% increment), and parenteral antibiotics increased from 4.02 DID to 5.15 DID (28.05% increment). In 2020, both oral and parenteral antibiotics declined against 2019, with the reduction of 25.38% and 30.64%, respectively. The use proportion of parenteral antibiotics increased from 31.08% in 2018 to 35.65% in 2019, and slightly declined in 2020 (33.99%). Central regions demonstrated the higher use proportion of parenteral antibiotics (39.2%) than eastern (30.1%) and western regions (35.0%).

**TABLE 2:** Antibiotic consumption in DID in different regions of China from 2018 to 2020.

Region	Route of administration	2018	2019	2020
Overall	Total	12.94	14.45	10.51
	Oral	8.92	9.30	6.94
	Parenteral	4.02	5.15	3.57
	Parenteral’s ratio (%)	31.08	35.65	33.99
Eastern China	Total	14.97	16.64	12.09
	Oral	10.95	11.19	8.45
	Parenteral	4.01	5.45	3.64
	Parenteral’s ratio (%)	26.82	32.76	30.10
Central China	Total	11.39	13.24	9.46
	Oral	7.17	7.90	5.76
	Parenteral	4.21	5.34	3.70
	Parenteral’s ratio (%)	37.00	40.35	39.15
Western China	Total	11.52	12.11	9.22
	Oral	7.81	7.89	5.99
	Parenteral	3.71	4.21	3.22
	Parenteral’s ratio (%)	32.23	34.79	34.97

Data of 24 provinces were involved. DID, defined daily doses per 1,000 inhabitants per day.

During the study period, the most commonly used antibiotic class in China were J01C (beta-lactam antibacterials and penicillins) and J01D (other beta-lactam antibacterials), followed by J01F (macrolides, lincosamides, and streptogramins) and J01M (quinolone antibacterials). The use proportion of J01C showed an upward trend from 28.11% in 2018 to 32.02% in 2020, while J01D showed an downward trend from 30.41% in 2018 to 28.86% in 2020. In 2020, J01C was the most consumed classes in central (37.73%) and western (38.48%) regions, while J01D ranked first in eastern regions (33.07%) (Table 3).

**TABLE 3:** Antibiotic consumption by ATC classification in different regions in China during 2018–2020.

Region	Categories	2018		2019		2020	
		DID	%	DID	%	DID	%
Overall	J01C_Penicillins	3.64	28.11	4.57	31.62	3.37	32.02
	J01D_Cephalosporins	3.94	30.41	4.28	29.64	3.03	28.86
	J01F_Macrolides and lincosamides	2.39	18.49	2.51	17.34	1.61	15.33
	J01M_Quinolones	1.51	11.69	1.65	11.42	1.34	12.73
	Other antibiotics	1.46	11.31	1.44	9.97	1.16	11.07
Eastern China	J01C_Penicillins	3.13	20.93	4.27	25.68	3.10	25.64
	J01D_Cephalosporins	5.23	34.91	5.57	33.49	4.00	33.07
	J01F_Macrolides and lincosamides	3.05	20.40	3.13	18.81	1.94	16.06
	J01M_Quinolones	1.94	12.96	2.06	12.38	1.63	13.53
	Other antibiotics	1.62	10.79	1.60	9.64	1.42	11.71
Central China	J01C_Penicillins	3.98	34.94	5.06	38.19	3.57	37.73
	J01D_Cephalosporins	2.92	25.63	3.37	25.45	2.31	24.43
	J01F_Macrolides and lincosamides	2.06	18.06	2.21	16.70	1.53	16.21
	J01M_Quinolones	1.21	10.65	1.39	10.48	1.14	12.06
	Other antibiotics	1.22	10.72	1.22	9.19	0.91	9.57
Western China	J01C_Penicillins	4.08	35.37	4.33	35.77	3.55	38.48
	J01D_Cephalosporins	3.07	26.67	3.25	26.83	2.37	25.74
	J01F_Macrolides and lincosamides	1.63	14.18	1.75	14.48	1.09	11.87
	J01M_Quinolones	1.16	10.10	1.28	10.54	1.09	11.84
	Other antibiotics	1.58	13.67	1.50	12.38	1.11	12.07

Note: Data of 24 provinces were involved. DID, defined daily doses per 1,000 inhabitants per day.

**FIGURE 2:**
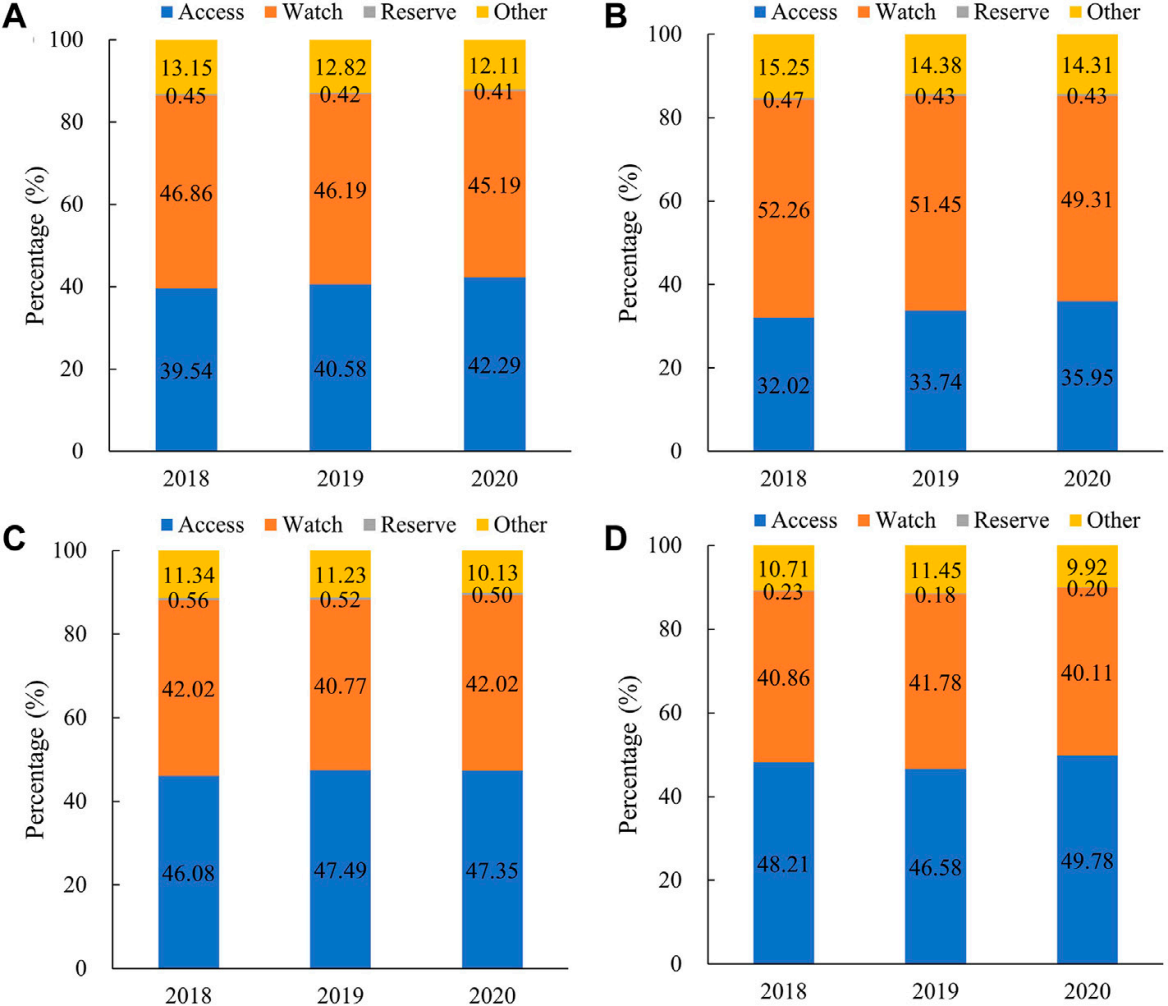
Proportional consumption (%) of antibiotics used in medical institutions by AWaRe categorization in China, 2018–2020. **(A)** Overall, **(B)** eastern China, **(C)** central China, and**(D)** western China. Note: Data of 24 provinces were involved.

Our study showed that the proportion of Access category antibiotics increased consistently between 2018 and 2020, from 39.54% to 42.29%. The antibiotics consumed in the Watch category had the largest proportion of 46.86% (2018), 46.19% (2019), and 45.19% (2020). In 2020, the use proportion of the Access category ranked first in central (47.35%) and western (49.78%) regions, while the Watch category was the most consumed in eastern regions (Figure 2).

### 3.2 Antibiotic Use by the Type of Medical Institution

**FIGURE 3:**
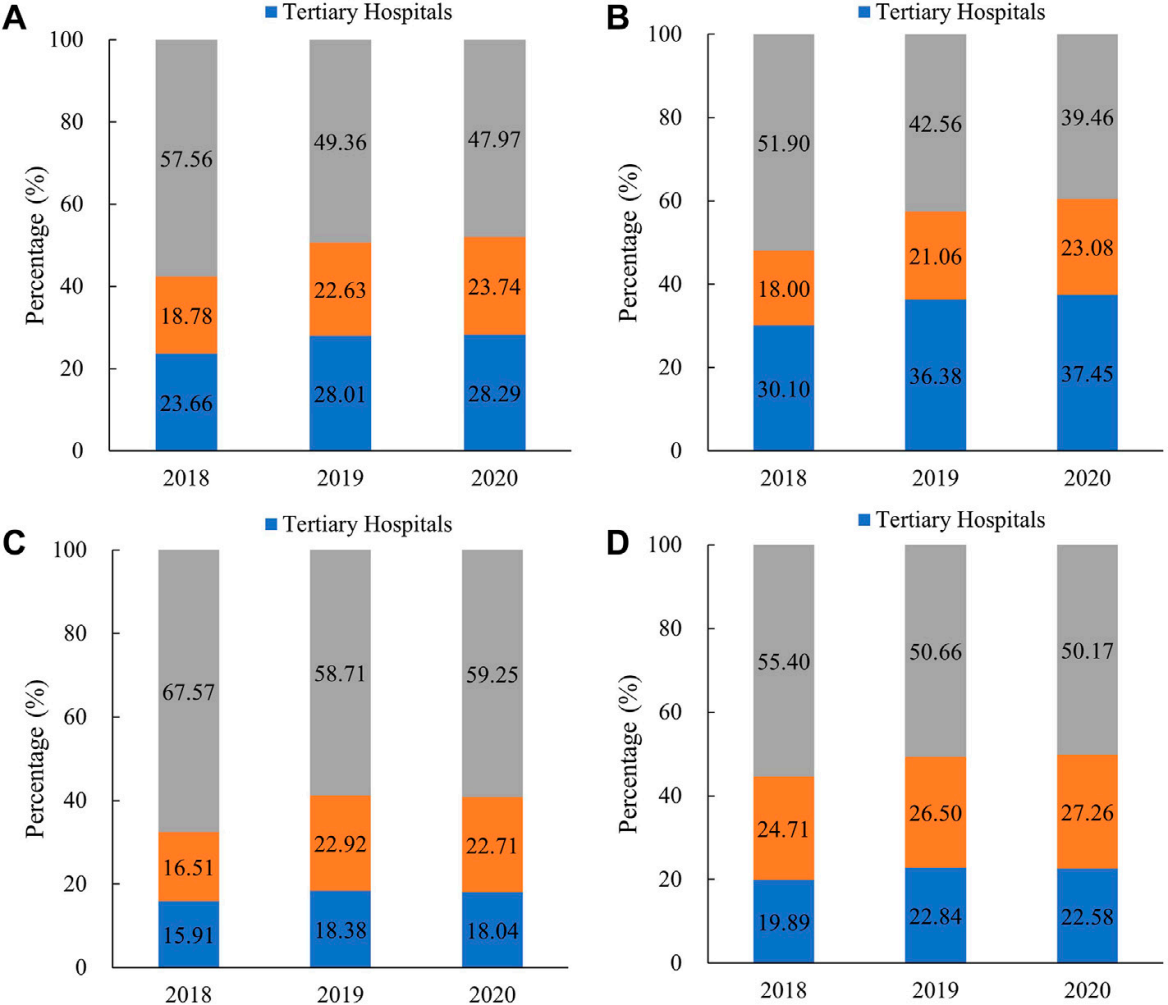
Proportional consumption (%) of antibiotics utilized in different medical institutions in China, 2018–2020. **(A)** Overall, **(B)** eastern China, **(C)** central China, and**(D)** western China. Note: Data of 24 provinces were involved.

Antibiotics consumed in tertiary and secondary hospitals increased by 29.96% and 32.34% from 2018 to 2019, while in PHCs, it decreased by 5.84%. Compared with 2019, 2020 consumption showed a great decline in tertiary hospitals (27.94%), secondary hospitals (25.16%), and PHCs (30.66%). Between 2018 and 2020, the proportion of antibiotics consumed in PHCs showed a downward trend from 57.56% to 47.97%, especially in eastern regions (from 51.90% to 39.46%). In 2020, tertiary hospitals, secondary hospitals, and PHCs shared 28.29%, 23.74%, and 47.97% of the total antibiotic consumption, respectively. Compared with the PHCs’ share in eastern regions (39.46%), antibiotics were far more consumed in PHCs in central (59.25%) and western (50.17%) regions in 2020 (Figure 3).

**FIGURE 4:**
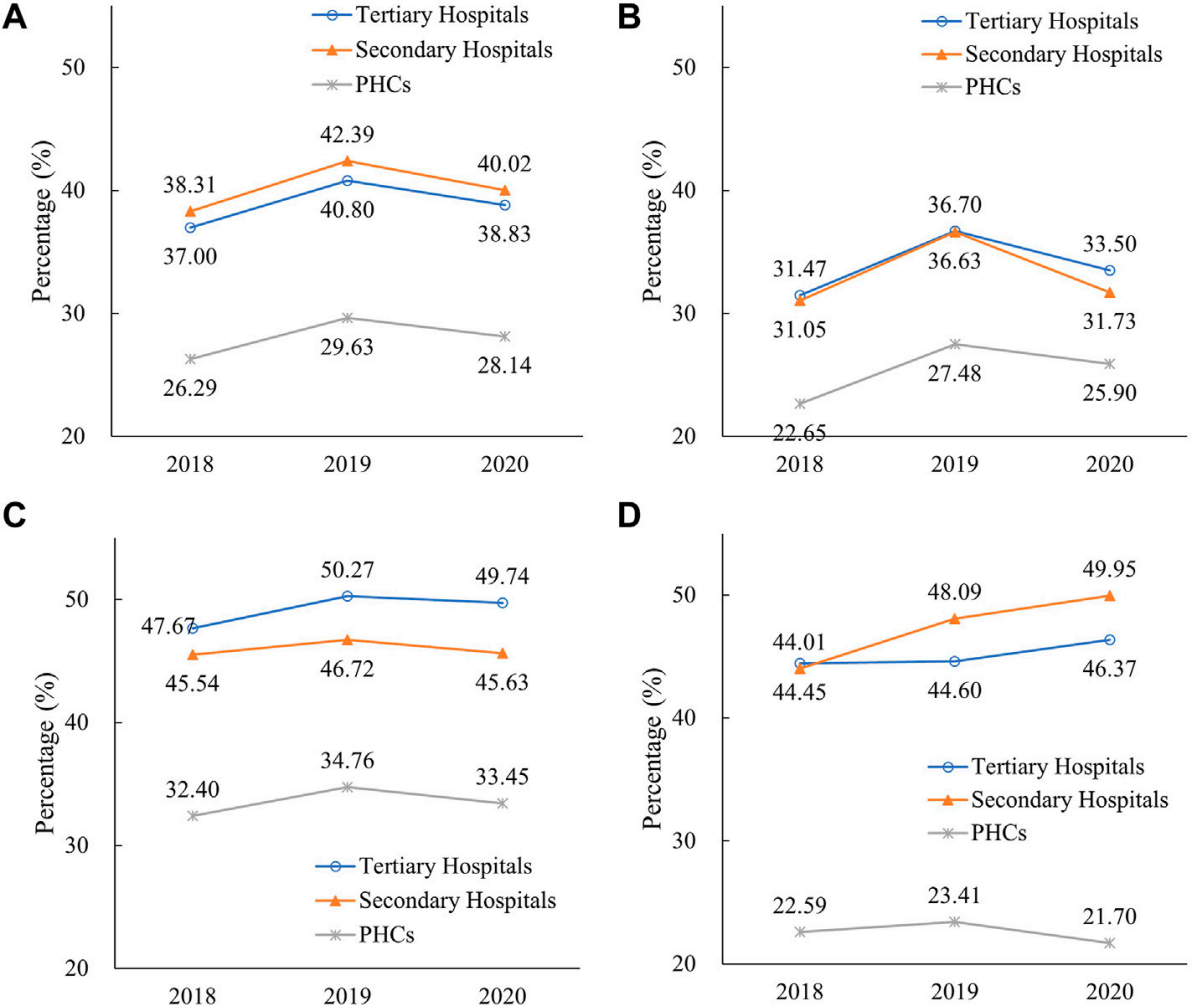
Utilization proportion of parenteral antibiotics in different medical institutions in China, 2018–2020. **(A)** Overall, **(B)** eastern China, **(C)** central China, and**(D)** western China. Note: Data of 24 provinces were involved. PHCs, primary healthcare centers.

**FIGURE 5:**
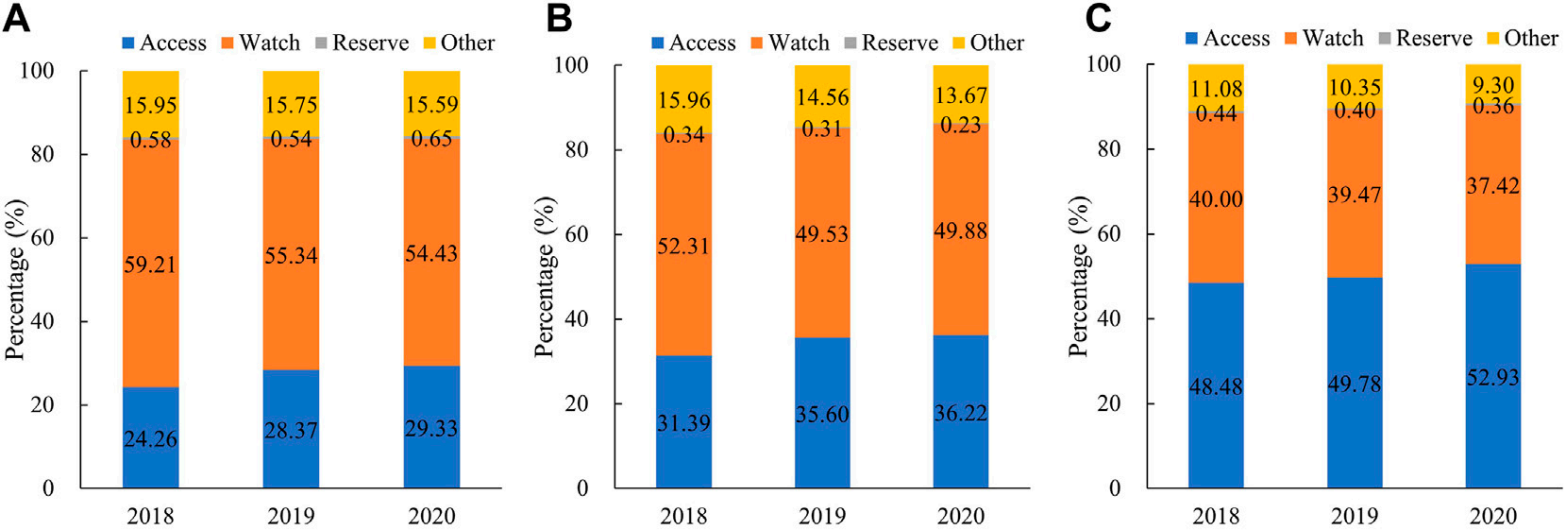
Proportional consumption (%) of antibiotics by AWaRe categorization in different medical institutions in China, 2018–2020. **(A)** Tertiary hospitals, **(B)** secondary hospitals, and**(C)** PHCs. Note: Data of 24 provinces were involved. PHCs, primary healthcare centers.

As shown in Figure 4, the use proportion of parenteral antibiotics increased in tertiary hospitals (3.80 percentage points), secondary hospitals (4.08 percentage points), and PHCs (3.34 percentage points) between 2018 and 2019, whereas it decreased to 38.83%, 40.02%, and 28.14% respectively, in 2020. In 2020, the parenteral proportion in tertiary (38.83%) and secondary (40.2%) hospitals was higher than that in PHCs (28.14%). In PHCs, the use proportion of parenteral antibiotics ranked as follows: central (33.45%) > eastern (25.90%) > western (21.70%) regions. In tertiary and secondary hospitals, central and western regions showed the higher proportion of parenteral antibiotic consumption. As for the consumption by the AWaRe category (Figure 5), Access category proportion increased steadily between 2018 and 2020 in tertiary hospitals (from 24.26% to 29.33%), secondary hospitals (from 31.39% to 36.22%), and PHCs (from 48.48% to 52.92%).

### 3.3 Change of Antibiotic Use Under the COVID-19 Pandemic

**FIGURE 6:**
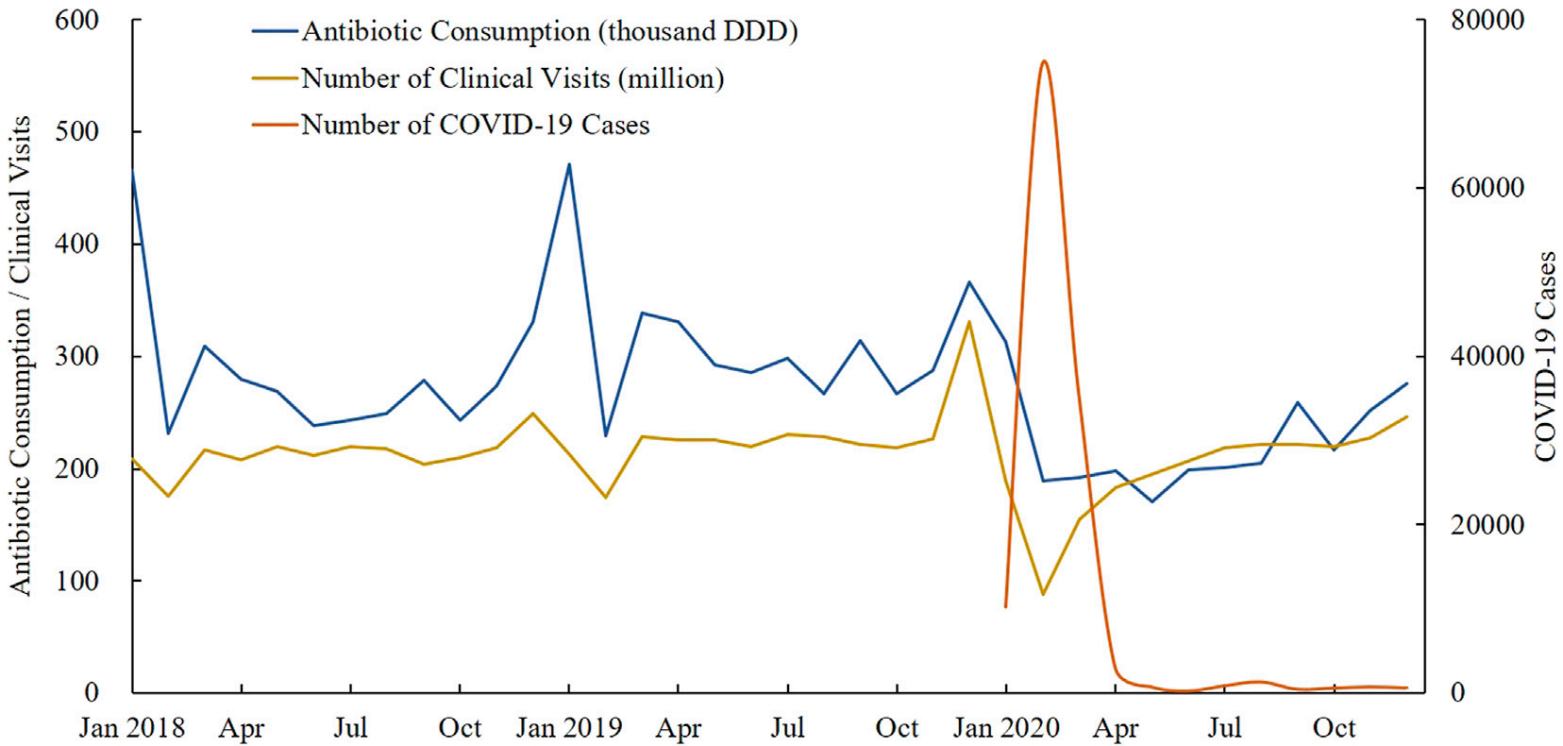
Monthly trend of antibiotic consumption, clinical visits, and number of COVID-19 cases under treatment between January 2018 and December 2020. Note: Data of 24 provinces were involved. DDD, defined daily doses.

Figure 6 outlined the monthly trend of antibiotic consumption between January 2018 and December 2020. In January 2020, with the outbreak of the COVID-19 pandemic in China, the number of diagnosed COVID-19 cases boosted and the hospitals’ clinic visits dropped sharply, followed by a significant decrease in antibiotic consumption.

As shown in Table 4, the results of regression analyses for medical service decline (Model I) showed that the total antibiotic consumption significantly decreased (*β* = −.14, *p* < .001) under the impact of medical service decline during the COVID-19 pandemic. Similar decline was observed in both hospitals (*β* = −.11, *p* < .001) and PHCs (*β* = −.17, *p* < .001), while the constituent ratio of antibiotics consumed in hospitals increased prominently (*β* = .02, *p* = .024) after COVID-19. As for the administration route, the consumption of both oral (*β* = −.15, *p* < .001) and parenteral (*β* = −.11, *p* < 0.001) antibiotics reduced, while no significant change of their constituent ratio was found (*β* = .02, *p* = .112).

**TABLE 4:** Generalized linear regression models on the change of antibiotic utilization under the COVID-19 pandemic.

Outcomes	Models I			Models II		
	Coef.	*p*-value	95% CI	Coef.	*p*-value	95% CI
Total (thousand DDD)	−.14	**.000**	−.17 to −.11	−.13	**.000**	−.17 to −.09
Type of medical institution						
Hospitals (thousand DDD)	−.11	**.000**	−.15 to −.08	−.09	**.000**	−.14 to −.04
PHCs (thousand DDD)	−.17	**.000**	−.21 to −.13	−.17	**.000**	−.23 to −.12
Hospitals’ ratio (%)	.02	**.024**	−.00 to .04	.03	**.007**	−.01 to .05
Route of administration						
Oral (thousand DDD)	−.15	**.000**	−.19 to −.11	−.13	**.000**	−.18 to −.08
Parenteral (thousand DDD)	−.11	**.000**	−.15 to −.08	−.12	**.000**	−.17 to −.07
Parenteral’s ratio (%)	.02	.112	−.004 to .04	.01	.632	−.02 to .04
Hospitals’ utilization						
Oral (thousand DDD)	−.13	**.000**	−.18 to −.09	−.10	**.001**	−.16 to −.04
Parenteral (thousand DDD)	−.09	**.000**	−.13 to −.06	−.08	**.001**	−.14 to −.03
Parenteral’s ratio (%)	.71	.064	−.04 to 1.46	.43	.403	−.58 to 1.45
PHCs’ utilization						
Oral (thousand DDD)	−.17	**.000**	−.22 to −.13	−.17	**.000**	−.23 to −.11
Parenteral (thousand DDD)	−.17	**.000**	−.23 to −.12	−.20	**.000**	−.27 to −.14
Parenteral’s ratio (%)	−.004	.812	−.04 to .03	−.06	**.026**	−.11 to −.01
Oral antibiotics						
Hospitals’ ratio (%)	.02	.144	−.01 to .04	.02	.093	−.004 to .05
Parenteral antibiotics						
Hospitals’ ratio (%)	1.73	**.001**	−.75 to 2.71	2.59	**.000**	−1.26 to 3.91
ATC class						
J01C (thousand DDD)	−.13	**.000**	−.18 to −.09	−.10	**.001**	−.17 to −.04
J01D (thousand DDD)	−.14	**.000**	−.17 to −.11	−.16	**.000**	−.20 to −.11
J01F (thousand DDD)	−.17	**.000**	−.20 to −.13	−.17	**.000**	−.22 to −.13
J01M (thousand DDD)	−.10	**.000**	−.13 to −.07	−.06	**.003**	−.11 to −.02
Other (thousand DDD)	−.11	**.000**	−.15 to −.06	−.11	**.000**	−.16 to −.05
ATC classes’ proportion						
J01C (%)	−.15	.620	−.76 to .45	.30	.464	−.51 to 1.12
J01D (%)	.10	.628	−.31 to .51	−.39	.167	−.94 to .16
J01F (%)	−.04	**.001**	−.07 to −.02	−.04	**.004**	−.07 to −.01
J01M (%)	.03	**.000**	−.02 to .05	.05	**.000**	−.03 to .07
Other (%)	−.01	.498	−.03 to .01	−.04	**.021**	−.07 to −.01
Access category proportion						
Overall (%)	.35	.288	−.29 to .99	.45	.314	−.42 to 1.31
Hospitals (%)	.35	.249	−.25 to .95	.62	.132	−.19 to 1.42
PHCs (%)	.87	**.019**	−.15 to 1.60	1.00	**.045**	−.02 to 1.98

Note: Bold values indicate regression coefficients with statistical significance (*p*< .05). Coef., coefficient; CI, confidence interval; DDD, defined daily doses; PHCs, primary healthcare centers. Model I estimated the impact of medical services decline on antibiotic use (Supplementary Table S1). Model II estimated the impact of number of COVID-19 cases under treatment on antibiotic use.

Affected by the decrease of medical services during the COVID-19 pandemic, oral and parenteral antibiotics consumed in both hospitals and PHCs dropped significantly (all *p*-values < .001). No significant effects were observed in the constituent ratio of oral and parenteral antibiotics consumed in both hospital and PHCs (all *p*-values > .05). The constituent ratio of oral antibiotics between hospitals and PHCs showed no significant changes after COVID-19 (*β* = .02, *p* = .144), whereas the proportion of parenteral antibiotics increased significantly in the setting of hospitals (*β* = 1.73, *p* = .001). Significant reductions were observed in the antibiotic consumption of each ATC-3 class (all *p*-values < .001). The use proportion of J01F class (*β* = −.04, *p* = .001) declined and J01M increased (*β* = .03, *p* < .001). After the COVID-19 pandemic, the use proportion of Access category antibiotics increased significantly in the setting of PHCs (*β* = .87, *p* = .019), while no significant changes in the use proportion of Access category antibiotics were observed in all medical institutions (*β* = .35, *p* = .288) and hospitals (*β* = .35, *p* = .249).

The results of regression analyses for the number of COVID-19 cases under treatment (Model II in Table 4) demonstrated the generally consistent modeling results with those in Model I. In addition, significant correlation was observed between medical service decline and the monthly number of COVID-19 cases under treatment (*r* = 0.580, *p* = .045) taking twenty-four sample provinces as a whole, whereas no significant correlations were found in fourteen of the twenty-four provinces (all *p*-values>.05) (Supplementary Table S4).

## 4 Discussion

Our study quantified the antibiotic consumption in China’s public medical institutions in 2018–2020 by using procurement data from a nationwide database. The evidence derived from 9176 public hospitals and 39,029 PHCs in mainland China would be informing for healthcare providers, decision-makers, as well as the public. The generalized linear regression models revealed that antibiotics consumed in China’s public medical institutions significantly declined and the use pattern has changed under the impact of the COVID-19 pandemic.

This study found that the total antibiotic consumption increased between 2018 and 2019 in China’s public medical institutions, and dropped in 2020 mainly due to the impact of COVID-19. The antibiotics consumed in specific provinces reported in this study were generally consistent with those in several previous provincial-level studies (Yin et al., 2018; Song et al., 2020), which was also in accordance with a nationwide study on the consumption of J01F antibiotics (2.91 DID in 2017) (Liu et al., 2021). However, the present result was higher than that of a previous study based on the China Medicine Economic Information (CMEI) procurement data in 2011–2018, which reported the antibiotic consumption of 6.7 DID in 2018 (Wushouer et al., 2020). As the authors stated (Wushouer et al., 2020), this inconsistency might be attributed to the involved healthcare facility type and the CMEI sample coverage. Furthermore, the present study revealed significant regional differences regarding antibiotic utilization in China (Qu et al., 2018). The eastern regions consumed more antibiotics than the central and western regions, which is consistent with previous findings (Wushouer et al., 2017; Liu et al., 2021). This might be explained by the patient flow between provinces caused by unbalanced healthcare resource allocation. Since the eastern regions are more developed than the central and western regions, the abundant healthcare resources, especially the best ones, brought patients from other underdeveloped regions.

This study showed that parenteral antibiotics accounted for 1/3 of the total antibiotic consumption in China’s public medical institutions, which is much higher than the proportion in most European countries reported by previous studies (Coenen et al., 2009; Robertson et al., 2021), and the proportion was more prominent in central and western regions than that in eastern regions. We found that the use proportion of parenteral antibiotics reached 40% in secondary and tertiary hospitals, which is consistent with previous reports based on hospital samples (Wushouer et al., 2017; Wushouer et al., 2020). The high proportion of parenteral antibiotics has consistently been a prominent problem in China. In China, as injections are more expensive and can be charged for injection or infusion, service providers might be more inclined to prescribe parenteral antibiotics. In addition, Chinese patients often perceive injections as being powerful, fast-acting, and longer lasting than oral pills (Lin et al., 2016; Zhang et al., 2019), which might also boost the high proportion of parenteral antibiotics in China. The Chinese government has proposed the drug use principle of “No intramuscular injection if can oral, no intravenous infusion if can intramuscular” (General Office of the State Council, 2018), and the use of parenteral antibiotics in Chinese medical institutions was monitored and assessed (National Health Commission, 2015), such as “the proportion of intravenous infusion of antibiotics.” However, according to the present finding, there is still much room for the decrease of parenteral antibiotics’ use proportion, especially in central and western regions, and in the healthcare settings of secondary and tertiary hospitals. In the future, the administration of parenteral antibiotics’ utilization should be strengthened in corresponding regions and relevant healthcare settings.

The AWaRe category was proposed by the WHO in the context of a comprehensive review of the optimal antibiotic choices for many common infectious syndromes in adults and children. The WHO set up a global target of greater than 60% use proportion of Access category antibiotics to reduce AMR (WHO, 2019). This study found that the proportion of Access category antibiotics consumed in China (42%) is far from WHO’s target and is much lower than that in Europe (57.9%) (Robertson et al., 2021). What is more, this ratio was even lower in eastern regions (36%), and the healthcare settings of secondary and tertiary hospitals (30%, 36%). Thus, to reach WHO’s global target, policy interventions at “high-risk” healthcare settings and key areas might make sense.

In this study, we explored the impact of the COVID-19 pandemic on antibiotic consumption in China’s public medical institutions, by taking medical services decline as the main intervention factor related to the pandemic. Regression analysis revealed that the shock of COVID-19 led to significant declines in antibiotic use in the healthcare settings of both hospitals and PHCs in China, which might be explained by China’s epidemic prevention and control strategies. With the outbreak of the epidemic, the leading group specific to COVID-19 was established immediately under the decision of the Communist Party of China Central Committee, to make unified leadership and command, and to deploy the whole nation’s epidemic prevention and control work (Zhang, 2021). A series of isolation and protection measures were introduced in succession, such as the requirement of “Isolation at home,” and the advocating of “Do not go to hospitals unless indeed necessary.” These measures to a large extent diminished the number of outpatient visits, hospitalizations, and surgeries (Wang et al., 2021); correspondingly, antibiotics consumed in public hospitals dropped. By adjusting the provincial COVID-19 cases, this study found that the modeling results of each outcome variable were generally consistent with those before adjustment. The results indicated that the impact of the COVID-19 pandemic on the population-level antibiotic consumption in China might be mainly attributed to the decline in medical services caused by epidemic prevention and control measures, rather than the treatment of COVID-19 cases. The Chinese government attached great importance to epidemic prevention prior to diagnosed cases, such as the popularity of wearing masks, nucleic acid tests for all residents, and travel controls, rather than just focusing on the treatment of diagnosed COVID-19 cases. Thus, there was no significant correlation between the number of provincial COVID-19 cases and the decline in medical services, which also supported the previous finding to a certain extent. However, relevant studies at the individual level might be needed to generate more explicit evidence.

The decline of antibiotic consumption in public medical institutions, on the one hand, might be attributed to the transfer of some medication demands to retail pharmacies during the epidemic (Ministry of Commerce of the PRC, 2021); on the other hand, it might be related to the decrease of unnecessary medical treatment and medication, as well as the reduced influenza incidence owe to COVID-19-related population-wide preventive measures, such as wear masks, maintain social distance, and diligent hand washing (Beijing Municipal Health Commission, 2020). Moreover, we found that COVID-19-caused reductions in antibiotic use were more prominent in the settings of PHCs than hospitals; this might be explained by the insufficient service capacity of PHCs which was even more prominent under COVID-19 shocks (Hao et al., 2020; Wang, 2021).

This study observed significant decline in antibiotic consumption in the healthcare settings of tertiary hospitals (27.94%), secondary hospitals (25.16%), and PHCs (30.66%) in China, which is far more prominent than the decline in EU/EEA countries (18.3% in primary care sector and 4.5% in hospital sector), especially in the healthcare setting of hospitals (ECDC, 2021). It may be related to China’s implementation of stricter prevention and control measures during the COVID-19 pandemic; people’s medical service contact was greatly restricted when compared with China’s flexible and ready access to hospital services before the pandemic. In addition, China is yet to establish an effective hierarchical diagnosis and treatment system (Shen and Du, 2019), and about 50% of the antibiotics were consumed in hospitals rather than in primary care settings, which is quite different with EU/EEA countries that 90% of antibiotics were used in communities (ECDC, 2021). This may be another reason why antibiotic use in Chinese hospitals dropped more sharply than that in EU/EEA countries during the epidemic.

Under the impact of COVID-19, no significant changes were observed in the constituent ratio of oral and parenteral antibiotics; however, we found that a certain proportion of parenteral antibiotic use flowed from PHCs to hospitals. It is known that the overuse of injections contributed to increased adverse drug reactions (Li, 2019), which undoubtedly would make matter worse for the PHCs with relatively weak service capacity. Previous studies reported that the issue of high parenteral antibiotic prescribing in PHCs was widespread in many regions of China (Jia et al., 2015; Bai et al., 2017). In this situation, the transferred consumption of parenteral antibiotics from PHCs to hospitals under COVID-19 might be an opportunity for improving antibiotic management in China. In addition, a prominent increase in the use proportion of Access category antibiotics was observed in PHCs in this study, which also released a positive signal regarding the use pattern changes of antibiotics in China’s primary healthcare settings.

To our knowledge, this is the nationwide antibiotic consumption study with the most up-to-date data (2018–2020) and the most complete sample coverage (9176 public hospitals and 39,029 PHCs) in China. The importance of this study lies in providing the latest evidence on antibiotic consumption in China’s public medical institutions under the context of the COVID-19 pandemic.

**FIGURE 7:**
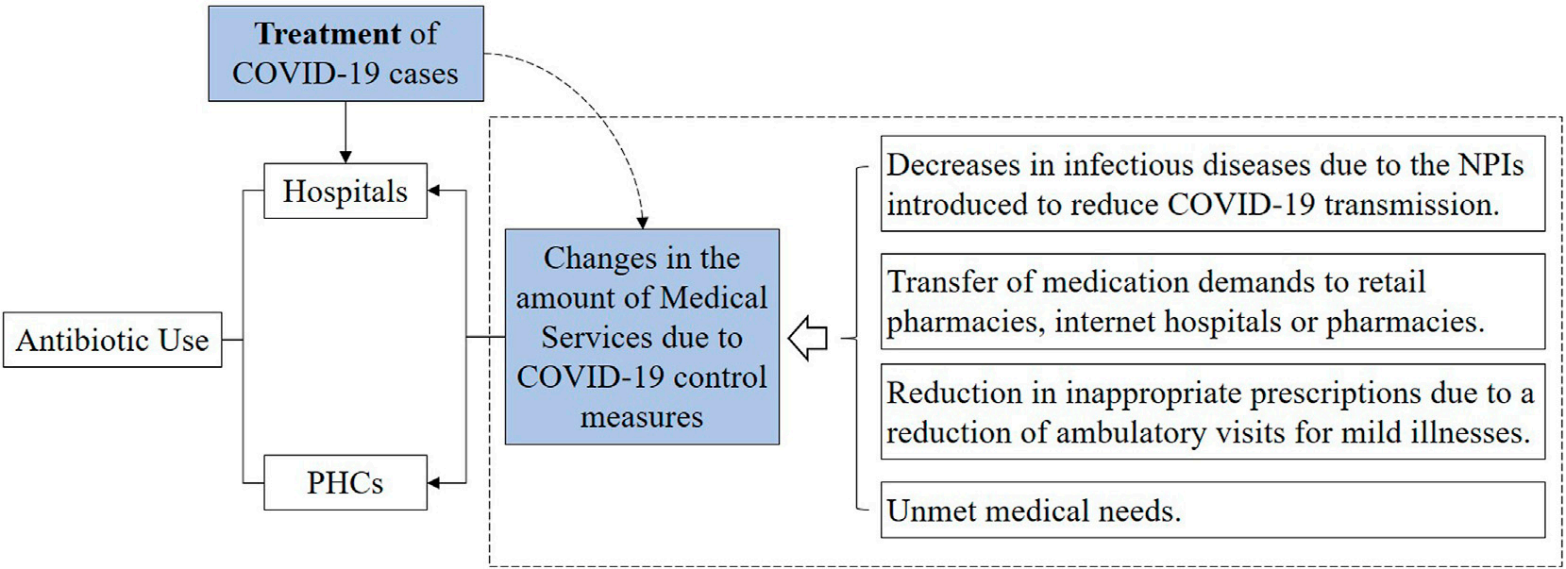
Influencing factors and their interaction relation of antibiotics use in China’s public medical institutions under the COVID-19 pandemic. Note: PHCs, primary healthcare centers; and NPIs, non-pharmaceutical interventions.

In China, the influence factors and their interaction relation of antibiotics use under the COVID-19 pandemic were becoming clear (Figure 7). The shock of COVID-19 in 2020 led to significant decline in antibiotic consumption around the world (Swedres-Svarm, 2020; Blix and Høye, 2021; Buehrle et al., 2021; ECDC, 2021; Gagliotti et al., 2021; Hogberg et al., 2021; King et al., 2021; Ng et al., 2021; Penalva et al., 2021; Sundhedsdatastyrelsen, 2021), and China is no exception. The decreases of antibiotic consumption during the COVID-19 pandemic might be due to the declined infectious diseases incidence caused by non-pharmaceutical interventions (NPIs) introduced to reduce COVID-19 transmission (Swedres-Svarm, 2020; Gagliotti et al., 2021; Penalva et al., 2021; Ullrich et al., 2021), as well as the reduction of medical service contact and the compression of irrational prescriptions during the pandemic lockdown. In the global context of antibiotic overuse, the COVID-19 pandemic might have certain positive contributions to the improvement of antibiotic management (Blix and Høye, 2021). In the future, policy-makers should pay attention to the change of antibiotic use under the COVID-19 pandemic, and give active guidance to seize the opportunity to improve the management of antibiotic use in China to a higher level. However, this study only described the change of China’s antibiotic use under the pandemic through population-level data; future individual-level studies are needed to identify the reasons for the change of antibiotic use, so as to promote management measure improvement.

This study also has a few limitations. First, the population denominator used for calculating DID in this study was determined under certain assumptions since we cannot access bed day data and the size of each health facility; meanwhile, the cross-provincial patient flow was not considered. Therefore, the estimation of the population covered by the included public health institutions might be biased. Second, this study analyzed provincial procurement data (i.e., population-level data) rather than the clinical usage of antibiotics. Although the method used is internationally accepted, the resulting DDD and DID data cannot be followed back to the demand of the individual patient. As patient- and prescriber-level data are not available, in this study, it is not possible to determine the direct causes behind the observed changes of antibiotic use during the COVID-19 pandemic, as well as whether there was any potential misuse of antibiotics during the treatment of COVID-19 cases. Third, since we cannot access the data of bed day and number of admissions of the sample medical institutions, this study failed to calculate the metric of DDD/100 bed days and DDD/100 admissions, which explain certain advantages of antibiotic consumption. Last, due to the lack of ATC-4 codes, this study analyzes data in ATC-3 rather than ATC-4, which might have limitations in information mining regarding antibiotic use.

## 5 Conclusion

This study revealed an increase in antibiotic consumption in China’s public medical institutions in 2018–2019, and a decline in 2020 mainly due to the impact of medical services decline caused by COVID-19 prevention and control measures. A consistent preference for penicillins and cephalosporins, and parenteral antibiotics, as well as low proportion of Access category antibiotics, was observed in China regardless of geographical regions or healthcare settings, which should be of concern. Under the impact of the COVID-19 pandemic, the total antibiotic consumption declined in China’s public medical institutions, and the utilization of antibiotics, especially for parenteral antibiotics, flowed from the healthcare setting of PHCs to hospitals, which may bring opportunities for the management of antibiotic use in China. In the future, policy-makers should pay attention to the change of antibiotic use under the COVID-19 pandemic and give active guidance to seize the opportunity to improve the management of antibiotic use in China to a higher level.

## Data Availability

The original contributions presented in the study are included in the article/Supplementary Material; further inquiries can be directed to the corresponding authors.
