# Supplementary material for: Antibiotic Use in China’s Public Healthcare Institutions During the COVID-19 Pandemic: An Analysis of Nationwide Procurement Data, 2018–2020
Source: Front Pharmacol. 2022 Feb 14;13:813213. doi: 10.3389/fphar.2022.813213 (PMC8882946; doi:10.3389/fphar.2022.813213)
Supplement: Supplementary file 1 [file DataSheet1.docx]

Supplementary Material

# Table S1. Decline range of medical services of sample provinces under COVID-19 pandemic.

| Province | Number of Clinical Visits | | |  | Growth rate (%) | | Decline range  of clinical visits  in 2020 | Group  code |
| --- | --- | --- | --- | --- | --- | --- | --- | --- |
|  | 2018 | 2019 | 2020 |  | 2019 | 2020 |  |  |
| Hubei | 9396.60 | 9363.70 | 4546.90 |  | -0.35 | -51.44 | ≥25% | 3 |
| Heilongjiang | 3499.10 | 3613.70 | 2109.20 |  | 3.28 | -41.63 | ≥25% | 3 |
| Shanghai | 7789.60 | 8438.00 | 5598.30 |  | 8.32 | -33.65 | ≥25% | 3 |
| Tianjin | 3688.40 | 3776.00 | 2559.00 |  | 2.38 | -32.23 | ≥25% | 3 |
| Jilin | 3103.90 | 3327.00 | 2315.10 |  | 7.19 | -30.41 | ≥25% | 3 |
| Liaoning | 5816.70 | 6173.30 | 4424.30 |  | 6.13 | -28.33 | ≥25% | 3 |
| Hainan | 1546.50 | 1666.20 | 1230.40 |  | 7.74 | -26.16 | ≥25% | 3 |
| Shaanxi | 5561.80 | 5930.10 | 4644.20 |  | 6.62 | -21.68 | 15%-25% | 2 |
| Inner Mongolia | 3000.00 | 3244.60 | 2548.10 |  | 8.15 | -21.47 | 15%-25% | 2 |
| Zhejiang | 19322.90 | 19917.20 | 15772.10 |  | 3.08 | -20.81 | 15%-25% | 2 |
| Guangxi | 7225.40 | 7710.50 | 6302.20 |  | 6.71 | -18.26 | 15%-25% | 2 |
| Henan | 15198.30 | 16617.30 | 13839.10 |  | 9.34 | -16.72 | 15%-25% | 2 |
| Shandong | 15055.60 | 15332.50 | 12860.90 |  | 1.84 | -16.12 | 15%-25% | 2 |
| Ningxia | 1326.40 | 1358.40 | 1142.30 |  | 2.41 | -15.91 | 15%-25% | 2 |
| Fujian | 6362.20 | 6525.50 | 5553.20 |  | 2.57 | -14.90 | <15% | 1 |
| Jiangxi | 4899.90 | 5635.20 | 4851.50 |  | 15.01 | -13.91 | <15% | 1 |
| Guizhou | 5143.90 | 5561.70 | 4903.50 |  | 8.12 | -11.83 | <15% | 1 |
| Shanxi | 3477.50 | 3678.80 | 3257.30 |  | 5.79 | -11.46 | <15% | 1 |
| Gansu | 2968.60 | 3201.10 | 2849.90 |  | 7.83 | -10.97 | <15% | 1 |
| Jiangsu | 17994.20 | 16372.40 | 14584.60 |  | -9.01 | -10.92 | <15% | 1 |
| Hebei | 8723.80 | 8709.30 | 7956.50 |  | -0.17 | -8.64 | <15% | 1 |
| Qinghai | 746.20 | 768.90 | 718.10 |  | 3.04 | -6.61 | <15% | 1 |
| Xinjiang | 3085.50 | 3676.40 | 3660.70 |  | 19.15 | -0.43 | <15% | 1 |
| Anhui | 7969.90 | 8511.60 | 8713.00 |  | 6.80 | 2.37 | <15% | 1 |

# Table S2. The monthly number of COVID-19 cases under treatment and the annual cumulative diagnosed cases in each province in 2020.

| Provinces | Number of COVID-19 cases under treatment in 2020 | | | | | | | | | | | | Cumulative diagnosed  cases in 2020 | Number of cases per  population (‰) |
| --- | --- | --- | --- | --- | --- | --- | --- | --- | --- | --- | --- | --- | --- | --- |
|  | Jan. | Feb. | Mar. | Apr. | May. | Jun. | Jul. | Aug. | Sep. | Oct. | Nov. | Dec. |  |  |
| Hubei | 7153 | 66492 | 33854 | 1609 | 7 | 3 | 0 | 4 | 0 | 0 | 9 | 4 | 68149 | 1.180 |
| Shanghai | 153 | 327 | 226 | 305 | 66 | 48 | 66 | 175 | 159 | 227 | 251 | 232 | 1516 | 0.061 |
| Zhejiang | 599 | 606 | 52 | 11 | 0 | 1 | 1 | 8 | 4 | 4 | 8 | 12 | 1306 | 0.020 |
| Henan | 422 | 1267 | 69 | 3 | 0 | 0 | 0 | 0 | 5 | 8 | 9 | 15 | 1299 | 0.013 |
| Anhui | 297 | 987 | 116 | 1 | 0 | 0 | 0 | 0 | 0 | 0 | 1 | 1 | 993 | 0.016 |
| Xinjiang | 18 | 76 | 11 | 0 | 0 | 0 | 563 | 810 | 34 | 54 | 78 | 0 | 980 | 0.038 |
| Heilongjiang | 80 | 478 | 141 | 468 | 345 | 2 | 0 | 1 | 0 | 1 | 1 | 15 | 964 | 0.030 |
| Jiangxi | 286 | 926 | 105 | 1 | 0 | 0 | 0 | 3 | 0 | 0 | 0 | 0 | 935 | 0.021 |
| Shandong | 202 | 754 | 337 | 27 | 13 | 5 | 7 | 38 | 17 | 17 | 12 | 14 | 862 | 0.008 |
| Jiangsu | 202 | 626 | 117 | 22 | 3 | 1 | 2 | 11 | 8 | 8 | 14 | 12 | 684 | 0.008 |
| Fujian | 144 | 296 | 101 | 58 | 4 | 6 | 6 | 19 | 40 | 46 | 77 | 69 | 513 | 0.012 |
| Shaanxi | 101 | 245 | 147 | 198 | 51 | 12 | 12 | 44 | 51 | 70 | 73 | 36 | 507 | 0.013 |
| Hebei | 96 | 317 | 34 | 12 | 4 | 21 | 9 | 16 | 13 | 9 | 8 | 1 | 373 | 0.005 |
| Inner Mongolia | 23 | 74 | 60 | 126 | 82 | 30 | 20 | 15 | 9 | 30 | 56 | 64 | 364 | 0.015 |
| Liaoning | 60 | 121 | 38 | 20 | 4 | 6 | 91 | 100 | 10 | 18 | 9 | 63 | 351 | 0.008 |
| Tianjin | 32 | 136 | 62 | 52 | 5 | 7 | 8 | 31 | 24 | 34 | 46 | 20 | 309 | 0.022 |
| Guangxi | 100 | 250 | 73 | 2 | 0 | 0 | 1 | 0 | 4 | 3 | 3 | 3 | 264 | 0.005 |
| Shanxi | 47 | 132 | 23 | 64 | 26 | 0 | 3 | 2 | 3 | 9 | 13 | 7 | 224 | 0.006 |
| Gansu | 35 | 91 | 52 | 12 | 5 | 30 | 19 | 2 | 3 | 7 | 12 | 1 | 182 | 0.007 |
| Hainan | 57 | 166 | 15 | 0 | 1 | 3 | 0 | 0 | 0 | 0 | 0 | 0 | 171 | 0.017 |
| Jilin | 17 | 93 | 22 | 18 | 53 | 9 | 2 | 2 | 0 | 0 | 0 | 0 | 157 | 0.007 |
| Guizhou | 29 | 144 | 33 | 1 | 0 | 0 | 0 | 0 | 0 | 0 | 0 | 0 | 147 | 0.004 |
| Ningxia | 26 | 73 | 6 | 0 | 0 | 0 | 0 | 0 | 0 | 0 | 0 | 0 | 75 | 0.010 |
| Qinghai | 9 | 18 | 0 | 0 | 0 | 0 | 0 | 0 | 0 | 0 | 0 | 0 | 18 | 0.003 |
| Total | 10188 | 74695 | 35694 | 3010 | 669 | 184 | 810 | 1281 | 384 | 545 | 680 | 569 | 81343 | 0.079 |

Monthly number of COVID-19 under treatment, refers to the number of COVID-19 cases still under treatment in each month of 2020 in each province, which is calculated as: diagnosed COVID-19 cases – cured and discharged cases (last month) – death cases (last month).

Data on COVID-19 cases were obtained from the National Health Commission website.

# Table S3. Results from modified park tests and box-cox tests to identify family distribution and link function in the generalized linear models for each outcome.

| Outcomes | Modified Park  Test | Family  Distribution | Box-cox Test | Link Function |
| --- | --- | --- | --- | --- |
| Total (thousand DDD) | 1.781 | gamma | 0.213 | log |
| Type of medical institution |  |  |  |  |
| Hospitals (thousand DDD) | 2.031 | gamma | 0.173 | log |
| PHCs (thousand DDD) | 1.812 | gamma | 0.169 | log |
| Hospitals’ ratio (%) | -1.779 | gaussian | 0.183 | log |
| Route of administration |  |  |  |  |
| Oral (thousand DDD) | 1.706 | gamma | 0.228 | log |
| Parenteral (thousand DDD) | 1.892 | gamma | 0.212 | log |
| Parenteral’s ratio (%) | 2.275 | gamma | 0.136 | log |
| Hospitals’ utilization |  |  |  |  |
| Oral (thousand DDD) | 2.038 | gamma | 0.134 | log |
| Parenteral (thousand DDD) | 1.938 | gamma | 0.249 | log |
| Parenteral’s ratio (%) | 0.005 | gaussian | 0.848 | identity |
| PHCs’ utilization |  |  |  |  |
| Oral (thousand DDD) | 1.565 | gamma | 0.160 | log |
| Parenteral (thousand DDD) | 2.190 | gamma | 0.214 | log |
| Parenteral’s ratio (%) | 0.407 | gaussian | 0.193 | log |
| Oral antibiotics |  |  |  |  |
| Hospitals’ ratio (%) | -1.597 | gaussian | 0.145 | log |
| Parenteral antibiotics |  |  |  |  |
| Hospitals’ ratio (%) | -1.433 | gaussian | 1.142 | identity |
| ATC class |  |  |  |  |
| J01C (thousand DDD) | 1.849 | gamma | 0.208 | log |
| J01D (thousand DDD) | 2.083 | gamma | 0.204 | log |
| J01F (thousand DDD) | 1.702 | gamma | 0.210 | log |
| J01M (thousand DDD) | 1.601 | gamma | 0.212 | log |
| Other (thousand DDD) | 1.651 | gamma | 0.239 | log |
| ATC classes’ proportion |  |  |  |  |
| J01C (%) | -0.051 | gaussian | 0.792 | identity |
| J01D (%) | 1.719 | gamma | 0.764 | identity |
| J01F (%) | -2.325 | gaussian | 0.126 | log |
| J01M (%) | -1.391 | gaussian | 0.244 | log |
| Other (%) | 6.240 | gamma | -0.162 | log |
| Access category proportion |  |  |  |  |
| Overall (%) | -1.275 | gaussian | 1.281 | identity |
| Hospitals (%) | 0.294 | gaussian | 0.692 | identity |
| PHCs (%) | -6.733 | gaussian | 1.493 | identity |

Note: Coefficients excerpted from modified Park tests: 0=Gaussian distribution (variance unrelated to the mean); 1=Poisson distribution (variance equal to the mean); 2=Gamma distribution (variance exceeding the mean); 3 =Inverse Gaussian distribution (or Wald distribution). Coefficients excerpted from Box-Cox tests: 0 =log link; 0.5=square root link; 1=identity link.

# Table S4. Spearman’s bivariate correlation coefficient between monthly medical services decline in 2020 and monthly number of COVID-19 cases under treatment in 2020.

| Provinces | n | Spearman *r* | *p*-value |
| --- | --- | --- | --- |
| Twenty-five provinces as a whole | 12 | 0.580 | 0.045* |
| Guizhou | 12 | 0.838 | 0.001** |
| Jilin | 12 | 0.817 | 0.001** |
| Tianjin | 12 | 0.816 | 0.001** |
| Hubei | 12 | 0.796 | 0.002** |
| Heilongjiang | 12 | 0.693 | 0.012* |
| Fujian | 12 | 0.643 | 0.024* |
| Hainan | 12 | 0.624 | 0.030* |
| Gansu | 12 | 0.620 | 0.032* |
| Anhui | 12 | 0.593 | 0.042* |
| Guangxi | 12 | 0.584 | 0.046* |
| Henan | 12 | 0.563 | 0.057 |
| Jiangxi | 12 | 0.534 | 0.074 |
| Shandong | 12 | 0.469 | 0.124 |
| Zhejiang | 12 | 0.448 | 0.144 |
| Inner Mongolia | 12 | 0.435 | 0.157 |
| Ningxia | 12 | 0.397 | 0.201 |
| Jiangsu | 12 | 0.368 | 0.239 |
| Hebei | 12 | 0.343 | 0.275 |
| Shaanxi | 12 | 0.320 | 0.310 |
| Qinghai | 12 | 0.298 | 0.346 |
| Shanxi | 12 | 0.292 | 0.358 |
| Xinjiang | 12 | 0.178 | 0.579 |
| Shanghai | 12 | 0.000 | 1.000 |
| Liaoning | 12 | 0.000 | 1.000 |

* *p*<0.05, ** *p*<0.01, *** *p*<0.001.

Monthly medical services decline in 2020, refers to the decrease of clinical visits in each month in 2020 compared with the same period in 2019 in each province. The declines were divided into three intervals: <10% (coded 1), 10%-20% (coded 2), and ≥20% (coded 3).

Monthly number of COVID-19 under treatment, refers to the number of COVID-19 cases still under treatment in each month of 2020 in each province, which is calculated as: diagnosed COVID-19 cases – cured and discharged cases (last month) – death cases (last month). It was divided into four intervals: no cases under treatment (coded 0), 1-49 cases (coded 1), 50-199 cases (coded 2), and ≥200 cases (coded 3).

Data on clinic visits and COVID-19 cases were obtained from the National Health Commission website.
